# The approaches, theories, models, frameworks, and methods in designing toolkits to support healthcare providers in health behaviour change: A scoping review protocol

**DOI:** 10.1371/journal.pone.0349867

**Published:** 2026-06-01

**Authors:** Jillian Scandiffio, Ashvene Sureshkumar, Gregory Feng, Dina Gaid, Marina Wasilewski, Nicole Woods, Sarah Donkers, Robert Simpson, Sarah Munce

**Affiliations:** 1 Rehabilitation Sciences Institute, University of Toronto, Toronto, Ontario, Canada; 2 St. Michael’s Hospital, Toronto, Ontario, Canada; 3 Toronto Rehabilitation Institute, University Health Network‌‌, Toronto, Ontario, Canada; 4 School of Rehabilitation Science, College of Medicine, University of Saskatchewan, Saskatoon, Saskatchewan, Canada; 5 The Institute for Education Research, University Health Network, Toronto, Ontario, Canada; 6 Institute of Health Policy, Management, and Evaluation, University of Toronto, Toronto, Ontario, Canada; 7 Holland Bloorview Kids Rehabilitation Hospital, Bloorview Research Institute‌‌, Toronto, Ontario, Canada; National Institute of Mental Health and Neurosciences: National Institute of Mental Health and Neuro Sciences, INDIA

## Abstract

**Objective:**

The scoping review aims to map the existing literature on the approaches, theories, frameworks, models and methods that have been used in the development of toolkits to support HCPs in health behaviour change.

**Introduction:**

Lifestyle behaviours are associated with a range of health outcomes, including comorbidities, health utilization, and quality of life. Healthcare providers (HCPs) are key figures in supporting various clinical populations to change these behaviours but are often hindered by external factors. It is important to develop evidence-based tools that support the needs of HCPs for health behaviour change. Toolkits inform and provide guidance for HCPs to bridge the evidence to practice gap in a variety of areas, including health behaviour. However, there is a lack of information as to how these toolkits should be developed. This review aims to fill this gap by mapping the existing literature on how toolkits to support HCPs in health behaviour change have been developed.

**Methods:**

This scoping review will follow the Joanna Briggs Institute (JBI) methodology and reporting will follow the Preferred Reporting Items for Systematic Reviews and Meta-analyses extension for scoping review (PRISMA-ScR). The search will be conducted across MEDLINE, Embase, Emcare, Cumulative Index to Nursing and Allied Health Literature (CINAHL), Web of Science, and Scopus. Screening and data extraction will be conducted independently and in duplicate, with any disagreements resolved through discussion and involvement of a third reviewer. Data will be extracted from studies using a tool developed by the reviewers, guided by the JBI template. For synthesis, quantitative data will be reported descriptively and a conventional content analysis will be undertaken for qualitative data.

**Inclusion Criteria:**

This scoping review will include studies published in peer-reviewed academic journals in English from any geographical area focusing on the development of a toolkit for HCPs involved in health behaviour change.

## Introduction

Lifestyle behaviours (e.g., smoking, physical activity, nutrition, etc.) are indicators of population health more broadly [1–4] and are associated with a range of health outcomes, including comorbidity (e.g., cardiovascular diseases, diabetes, cancer) [5,6], health service utilization [7–9], and quality of life [10]. Health care providers (HCPs) are key agents in supporting health behaviour change in a variety of clinical populations [11–13]. However, there is evidence to suggest that a HCP’s ability to address health behaviours in day-to-day care is hindered by multiple external factors, including, but not limited to, health system gaps, lack of confidence in counselling behaviour change, and lack of time [14].

It is important to develop user-friendly, evidence-based tools that support the needs of HCPs for health behaviour change. Toolkits are collections of adaptable resources to inform and provide guidance which are typically intended to bridge the evidence to practice gap, support implementation processes for adoption, and/or support sustained use of the intended change [15]. Toolkits can be used by themselves as a knowledge translation strategy or can be part of a larger multi-strategy intervention [16]. Toolkits have shown effectiveness in improving clinical outcomes (e.g., reduced patient falls, decreased hospital length of stay, improved diabetes management). [16–18] The definition of what encompasses a “toolkit” is somewhat heterogenous, as toolkits range greatly in modality, formatting and content and may include individual or combinations of documents such as timelines, educational materials, and assessment instruments [15,16]. Toolkits have demonstrated effectiveness [17,18] and the wide range of toolkit designs suggests that before a toolkit is developed and implemented there should be a solid understanding as to *how* it should be developed. An evidence-informed approach to toolkit design may improve engagement, uptake, and effectiveness.

A preliminary search of MEDLINE, the Cochrane Database of Systematic Reviews, Open Science Framework, and *JBI Evidence Synthesis* was conducted, and no published or currently underway reviews were found. While there have been scoping reviews on toolkits in healthcare [16,19], there is a lack of information regarding the development of these toolkits. Additionally, there have been recommendations for development of a toolkit in areas outside of health behaviour change (e.g., program evaluation toolkit) [20], but there is a lack of information regarding best practices for the development of toolkits to support HCPs in health behaviour change. Further, there is a lack of information regarding best practices for the development of toolkits to support HCPs in health behaviour change. Thus, there is a gap in understanding best practices for the development of a toolkit to support HCPs in addressing health behaviour change. By gaining a deeper understanding of the optimal approaches to toolkit development, we can better design future toolkits for HCPs. This review may act as a resource for investigators looking to develop future toolkits for HCPs in health behaviour change.

The objective of this scoping review is to map the existing literature on the approaches, theories, frameworks, models and methods that have been used in the development of toolkits to support HCPs in health behaviour change.

### Review question‌‌

What is the extent of the literature on the approaches, theories, models, frameworks, and methods that have been used to develop and evaluate toolkits to support HCPs for the specific purpose of health behaviour change in their patients?

### Inclusion criteria

#### Population.

The population of interest for this scoping review is health care providers (HCPs) who are aiming to change the health behaviours of their patients.

#### Concept.

The conceptual focus is on the development approach of toolkits to support healthcare providers in addressing lifestyle-related health behaviour change with their patients of any age with chronic conditions. Health behaviours will include negative health behaviours (smoking/tobacco use, recreational drug use, alcohol use), movement behaviours (e.g., physical activity, sedentary behaviour), sleep, and nutrition, based on the categories of health behaviours reported by Statistics Canada^1^. Chronic conditions will be based on the chronic conditions identified by Statistics Canada and the Public Health Agency of Canada, [21,22] with chronic conditions added as relevant (e.g., a rare chronic neurological condition not explicitly mentioned by the organizations but falling under a broader category of ‘neurological condition’). The study will be included if it 1) describes the approach, theory, framework, model, and/or method for designing toolkits to support HCPs in health behaviour change; and 2) the primary focus of the paper is on the toolkit design approach. We will include studies in which the study authors describe their intervention as a “tool” or a Boolean operator of this term (e.g., toolkit) but will exclude studies where the intervention is not referred to using this term (e.g., package, materials). The study will be excluded if the focus of the toolkit is on the change of health behaviour in HCPs and not patients (e.g., improved nutrition in medical residents).

#### Context.

Studies published in peer-reviewed academic journals in English from any geographical area focusing on the development of a toolkit for HCPs involved in health behaviour change will be included.

#### Types of sources.

This scoping review will consider all study designs, as long as it includes primary research data and they describe approaches for developing toolkits in detail.

### Methods

The proposed scoping review will be undertaken in accordance with the JBI methodology guidance for conducting scoping reviews [23]. The reporting of the protocol follows PRISMA-P and the scoping review reporting will follow Preferred Reporting Items for Systematic Reviews and Meta-analyses extension for scoping review (PRISMA-ScR) [24] guidelines (S1 File). The results of the search and the study inclusion process will be reported in full in the final scoping review and presented in a PRISMA-ScR flow diagram [24].

### Protocol and registration

The protocol was registered on Open Science Framework on November 22, 2024 (Registration doi: https://doi.org/10.17605/OSF.IO/BSTMY).

### Search strategy

The electronic database search strategy will be developed in collaboration with an academic librarian. A three-step search strategy will be utilized in this review. First, an initial limited search will be completed in Medline (Ovid) using a combination of subject headings and text words for the following concepts: toolkits, healthcare providers, health behaviour, models, theories, and frameworks. For this review, a model is defined as a simplification or specific aspect of a phenomenon, a theory is defined as “a set of analytical principles or statements designed to structure our observation, understanding, and explanation of the world”[25, p2], and a framework as an explanation of a phenomena through a structure, overview, outline, system, or plan of descriptive categories and the relationships between them [25].

Search terms and appropriate synonyms will be confirmed through team discussion. The Medline search will be peer-reviewed following Peer Review of Electronic Search Strategies (PRESS) guidelines [26]. The finalized Medline search will then be translated to the following additional databases: Embase (Ovid), Emcare (Ovid), CINAHL (EBSCOhost), Web of Science (Clarivate), and Scopus (S2 File). The reference list of all included sources of evidence will be screened for additional studies. Studies published in any language will be included. All studies published since database inception will be included. The reference list of all included sources of evidence will be screened for additional studies.

### Study/Source of evidence selection

Following the search, all identified citations will be collated and uploaded into EndNote20 (Clarivate Analytics, PA, USA) and Covidence, with duplicates removed. A pilot review of 100 of the titles will be screened by two reviewers to establish inter-rater reliability, with a Cohen’s Kappa of 0.8 indicating substantial agreement [27]. Once this threshold has been reached, titles and abstracts will then be screened by two or more independent reviewers for assessment against the inclusion criteria for the review. Potentially relevant sources will be retrieved in full in Covidence. The full text of selected citations will be assessed in detail against the inclusion criteria by two or more independent reviewers. Reasons for exclusion of sources of evidence at full text that do not meet the inclusion criteria will be recorded and reported in the scoping review. Any disagreements that arise between the reviewers at each stage of the selection process will be resolved through discussion and will consult a third party (another reviewer) if consensus cannot be achieved [23].

### Data extraction

Data will be extracted from full papers included in the scoping review by two or more independent reviewers using a data extraction tool developed by the reviewers, guided by the JBI template (S3 File) [28]. The data extracted will include authors, year of publication, origin/country of publication, country of toolkit development, study aims/purpose, HCP population (e.g., nurses, allied health professionals), patient chronic condition (e.g., cancer, multiple sclerosis), intersectional factors guided by the PROGRESS-Plus framework for considerations related to tailoring toolkits (e.g., age, gender, race/ethnicity), health behaviour, toolkit aim, toolkit componentry, toolkit development, evaluation and implementation process, models, frameworks and/or theories applied, knowledge users involved, outcomes, and recommendations provided/lessons learned.

A draft extraction form is provided (see S3 File). The draft data extraction tool will be modified and revised as necessary during the process of extracting data from each included evidence source. Modifications will be detailed in the scoping review. Any disagreements that arise between the reviewers will be resolved through discussion, or with an additional, senior reviewer. If appropriate, authors of papers will be contacted to request missing or additional data, where required. It is expected that data collection will be completed by July 2025.

### Data analysis and presentation

Quantitative data will be reported in simple descriptive terms in both tabular form and a narrative summary. A conventional content analysis [29] will be undertaken for qualitative data, with a simple descriptive synthesis of findings (e.g., barriers and facilitators to implementation). Barriers and facilitators will be identified from studies and will be deductively coded using the Theoretical Domains Framework (TDF) and reported according to TDF domains identified in studies, as this framework identifies factors to influence HCP behaviour [30]. It is expected that results of the study will be available by May 2026.

## Discussion

This review will provide useful information at a time of rise in prevalence of knowledge translation initiatives to support HCPs [30]. First, it will provide insight into the definition of a “toolkit” in health implementation research, as there is currently no consensus on what is included in a toolkit nor how a toolkit is intended to be used. This will contextualize a broad phenomenon and challenge implementation scientists to consider terminology of intervention materials for HCPs. Second, it will provide valuable information about the theories, models, and frameworks used in the development and evaluation of toolkits, thereby elucidating optimal approaches to designing and developing future toolkits for HCPs.

The results of this review will be disseminated through traditional knowledge mobilization approaches of publication in an academic journal and conference presentation. The results will be published in an appropriate, reputable peer-reviewed journal for health behaviour change and/or implementation science to reach HCPs, researchers, and other relevant knowledge users in the area. Further, by presenting findings at a conference aimed at relevant knowledge users nationally, the findings from this review can be disseminated to implementation scientists and those looking to develop materials to support HCPs in behaviour change. Further, and more specifically, the results of this review will help to inform the development of a toolkit to support HCPs in health-related lifestyle behaviour change in PwMS. The approaches, methods, theories, models, and frameworks identified in this review will inform strategies for the development and evaluation of the aforementioned toolkit.

This review has several strengths. The review will be high-quality and methodologically rigorous, being guided by JBI methodology guidance for scoping reviews and adhering to PRISMA-ScR guidelines for reporting. The search strategy will be developed by an academic librarian and peer reviewed by another academic librarian to ensure appropriate articles are captured. Additionally, screening and data extraction will be conducted in duplicate by independent reviewers. Further, this team has reviewers with a background in systematic literature syntheses, implementation science, toolkit development and health behaviour change.

There are some anticipated limitations of this review. This review will only include studies in English, which may limit our understanding of toolkit development in primarily non-English speaking countries. Further, this review will exclude grey literature, which may result in exclusion of literature related to the development of toolkits in non-academic settings. Finally, the review will not provide information regarding the methodological quality of the individual studies, instead providing an outline of the approaches, theories, frameworks, models, and methods that have been used in the development of toolkits to support HCPs in health behaviour change.

## Supporting information

S1 File PRISMA-ScR checklist. (DOCX)

S2 File EMBASE search strategy. (DOCX)

S3 File Data extraction instrument. (DOCX)

## References

[1] Statistics Canada. Health Behaviours. 2010.

[2] Seo B, Nan H, Monahan PO, Duszynski TJ, Thompson WR, Zollinger TW, et al. Association between US Residents’ Health Behavior and Good Health Status at the City Level. Transl J ACSM. 2024;9(2). doi: 10.1249/tjx.0000000000000258

[3] Mozaffarian D, Afshin A, Benowitz NL, Bittner V, Daniels SR, Franch HA, et al. Population approaches to improve diet, physical activity, and smoking habits: a scientific statement from the American Heart Association. Circulation. 2012;126(12):1514–63. doi: 10.1161/CIR.0b013e318260a20b 22907934 PMC3881293

[4] Short SE, Mollborn S. Social determinants and health behaviors: conceptual frames and empirical advances. Curr Opin Psychol. 2015;5:78–84. 26213711 10.1016/j.copsyc.2015.05.002 PMC4511598

[5] Spring B, Moller AC, Coons MJ. Multiple health behaviours: overview and implications. J Public Health (Oxf). 2012;34 Suppl 1(Suppl 1):i3-10. doi: 10.1093/pubmed/fdr111 22363028 PMC3284863

[6] Giannakou K, Kyprianidou M, Chrysostomou S, Christophi CA. Editorial: The associations of lifestyle factors and behaviors with multimorbidity. Front Public Health. 2023;11:1227381. doi: 10.3389/fpubh.2023.1227381 37441654 PMC10335786

[7] Tran B, Falster MO, Douglas K, Blyth F, Jorm LR. Health behaviours and potentially preventable hospitalisation: a prospective study of older Australian adults. PLoS One. 2014;9(4):e93111. doi: 10.1371/journal.pone.0093111 24691471 PMC3972201

[8] US Department of Health and Human Services OP. Foundation health measures: Determinants of health. In: Services UDHH. Washington, DC. 2017.

[9] Manuel DG, Bennett C, Perez R, Wilton AS, Rohit Dass A, Laporte A, et al. Burden of health behaviours and socioeconomic position on health care expenditure in Ontario. F1000Res. 2019;8:303. doi: 10.12688/f1000research.18205.2 31723417 PMC6844135

[10] Espinosa-Salas S, Gonzalez-Arias M. Behavior Modification for Lifestyle Improvement. StatPearls. Treasure Island (FL). 2024. 37276296

[11] Cunningham C, O’Sullivan R. Healthcare Professionals’ Application and Integration of Physical Activity in Routine Practice with Older Adults: A Qualitative Study. Int J Environ Res Public Health. 2021;18(21):11222. doi: 10.3390/ijerph182111222 34769742 PMC8582732

[12] Pipe AL, Evans W, Papadakis S. Smoking cessation: health system challenges and opportunities. Tob Control. 2022;31(2):340–7. doi: 10.1136/tobaccocontrol-2021-056575 35241609

[13] Kraef C, Wood B, von Philipsborn P, Singh S, Peterson SS, Kallestrup P. Primary health care and nutrition. Bull World Health Organ. 2020;98(12):886–93. doi: 10.2471/BLT.20.251413 33293749 PMC7716102

[14] Vallis M, Lee-Baggley D, Sampalli T, Ryer A, Ryan-Carson S, Kumanan K, et al. Equipping providers with principles, knowledge and skills to successfully integrate behaviour change counselling into practice: a primary healthcare framework. Public Health. 2018;154:70–8. doi: 10.1016/j.puhe.2017.10.022 29216495

[15] Thoele K, Ferren M, Moffat L, Keen A, Newhouse R. Development and use of a toolkit to facilitate implementation of an evidence-based intervention: a descriptive case study. Implement Sci Commun. 2020;1:86. doi: 10.1186/s43058-020-00081-x 33043301 PMC7539511

[16] Yamada J, Shorkey A, Barwick M, Widger K, Stevens BJ. The effectiveness of toolkits as knowledge translation strategies for integrating evidence into clinical care: a systematic review. BMJ Open. 2015;5(4):e006808. doi: 10.1136/bmjopen-2014-006808 25869686 PMC4401869

[17] Dykes PC, Duckworth M, Cunningham S, Dubois S, Driscoll M, Feliciano Z, et al. Pilot Testing Fall TIPS (Tailoring Interventions for Patient Safety): a Patient-Centered Fall Prevention Toolkit. Jt Comm J Qual Patient Saf. 2017;43(8):403–13. doi: 10.1016/j.jcjq.2017.05.002 28738986

[18] Helmle KE, Chacko S, Chan T, Drake A, Edwards AL, Moore GE, et al. Knowledge Translation to Optimize Adult Inpatient Glycemic Management With Basal Bolus Insulin Therapy and Improve Patient Outcomes. Can J Diabetes. 2018;42(5):505-513.e1. doi: 10.1016/j.jcjd.2017.12.010 29555341

[19] Colquhoun HL, Squires JE, Kolehmainen N, Fraser C, Grimshaw JM. Methods for designing interventions to change healthcare professionals’ behaviour: a systematic review. Implement Sci. 2017;12(1):30. doi: 10.1186/s13012-017-0560-5 28259168 PMC5336662

[20] Davidson RJ, Kaszniak AW. Conceptual and methodological issues in research on mindfulness and meditation. Am Psychol. 2015;70(7):581–92. doi: 10.1037/a0039512 26436310 PMC4627495

[21] Statistics Canada. Health of Canadians Health Outcomes. 2024. https://www150.statcan.gc.ca/n1/pub/82-570-x/2024001/section2-eng.htm#a3

[22] Public Health Agency of Canada. Chronic Diseases. 2024. https://www.canada.ca/en/public-health/services/chronic-diseases.html

[23] Peters MDJ, Godfrey CM, McInerney P, Munn Z, Tricco AC, Khalil H. Chapter 11: Scoping Reviews (2020 version). In: Aromataris E, Munn Z. JBI Manual for Evidence Synthesis. JBI. 2020.

[24] Tricco AC, Lillie E, Zarin W, O’Brien KK, Colquhoun H, Levac D, et al. PRISMA Extension for Scoping Reviews (PRISMA-ScR): Checklist and Explanation. Ann Intern Med. 2018;169(7):467–73. doi: 10.7326/M18-0850 30178033

[25] Nilsen P. Making sense of implementation theories, models and frameworks. Implement Sci. 2015;10:53. doi: 10.1186/s13012-015-0242-0 25895742 PMC4406164

[26] McGowan J, Sampson M, Salzwedel DM, Cogo E, Foerster V, Lefebvre C. PRESS Peer Review of Electronic Search Strategies: 2015 Guideline Statement. J Clin Epidemiol. 2016;75:40–6. 27005575 10.1016/j.jclinepi.2016.01.021

[27] McHugh ML. Interrater reliability: the kappa statistic. Biochem Med (Zagreb). 2012;22(3):276–82. doi: 10.11613/bm.2012.031 23092060 PMC3900052

[28] Peters M, Godfrey CM, McInerney P, Baldini Soares C, Khalil H, Parker D. Appendix 11.1 JBI template source of evidence details, characteristics and results extraction instrument. In: Aromataris E, Munn Z. JBI Manual for Evidence Synthesis. JBI. 2020.

[29] Hsieh H-F, Shannon SE. Three approaches to qualitative content analysis. Qual Health Res. 2005;15(9):1277–88. doi: 10.1177/1049732305276687 16204405

[30] King O, West E, Alston L, Beks H, Callisaya M, Huggins CE, et al. Models and approaches for building knowledge translation capacity and capability in health services: a scoping review. Implement Sci. 2024;19(1):7. doi: 10.1186/s13012-024-01336-0 38287351 PMC10823722

