# Supplementary material for: The approaches, theories, models, frameworks, and methods in designing toolkits to support healthcare providers in health behaviour change: A scoping review protocol
Source: PLoS One. 2026 Jun 1;21(6):e0349867. doi: 10.1371/journal.pone.0349867 (PMC13225628; doi:10.1371/journal.pone.0349867)
Supplement: S2 File — (DOCX) [file pone.0349867.s002.docx]

**Supplementary file 2 – Search strategy**

**Embase Classic - Embase <1947 to 2024 November 20>**

| 1 | tool*.ti. | 165468 |
| --- | --- | --- |
| 2 | "Exercise and Depression Toolkit".ti,ab. | 3 |
| 3 | "5As obesity tools".ti,ab. | 0 |
| 4 | quality assessment tool/ or "tool use"/ | 4893 |
| 5 | 1 or 2 or 3 or 4 | 169078 |
| 6 | translational research/ or motivational interviewing/ or transtheoretical model/ | 32422 |
| 7 | "Theoretical Domains framework".ti,ab,kw. | 2125 |
| 8 | Social Learning Theory/ or "Theory of Planned Behavior"/ | 3529 |
| 9 | Agree II instrument.ti,ab,kw. | 831 |
| 10 | ((knowledge or research) adj2 translat*).ti,ab,kw. | 35295 |
| 11 | "Knowledge To Action framework".ti,ab,kw. | 207 |
| 12 | "Theory of Reasoned Action"/ or Social Cognitive Theory/ or co-design*.tw,kw. | 7959 |
| 13 | ("social cognitive theory" or "transtheoretical model" or "self determination theory" or "motivational interviewing" or "theory of planned behavio?r" or "social learning theory" or "theory of reasoned action" or "stages of change" or "health belief model" or "behavio?r change wheel" or "personalization theory" or "social practice theory" or nudg* or "stages of change").tw,kw. | 32333 |
| 14 | or/6-13 | 91679 |
| 15 | health education/ or behavior change/ or behavior modification/ or health promotion/ or healthy lifestyle/ or exp health behavior/ | 778880 |
| 16 | ((health or lifestyle or behavio?r*) adj2 (interv* or chang* or modif* or prevent* or promot* or model* or framework* or method* or approach*)).tw,kw. | 482027 |
| 17 | 15 or 16 | 1121958 |
| 18 | exercise/ or physical activity/ or sleep/ or smoking/ or alcoholism/ or heavy drinking/ or nutrition/ or diet/ or body weight management/ or obesity/ or drinking behavior/ | 2109961 |
| 19 | (weight or obese or obesity or obeseness or over-weight or sleep* or sedentary or exercis* or "physical activ*" or smoking or nutrition or alcohol* or "heavy drinking" or diet* or lifestyle).tw,kw. | 4501032 |
| 20 | 18 or 19 | 4960124 |
| 21 | (("knowledge translation" or interven* or framework* or method* or approach*) adj1 (health* adj2 (behavio?r or chang* or modif* or promot* or lifestyle or prevent*))).tw. | 6543 |
| 22 | health care personnel/ | 270914 |
| 23 | ((health-care or hospital or medical or allied-health) adj1 (practitioner* or professional* or provider* or personnel or support worker*)).tw,kw. | 163813 |
| 24 | health practitioner/ | 64945 |
| 25 | paramedical personnel/ | 18063 |
| 26 | (para-medical adj1 (personnel or assistant* or professional* or manpower)).tw,kw. | 71 |
| 27 | 22 or 23 or 24 or 25 or 26 | 460447 |
| 28 | 5 and 14 and 17 and 20 and 27 | 21 |
| 29 | 5 and 14 and 17 and 20 | 111 |
| 30 | 5 and 17 and 20 | 2133 |
| 31 | 5 and 17 and 20 and 27 | 198 |
| 32 | 5 and 14 and 20 | 225 |
| 33 | 14 and 17 and 20 and 27 | 931 |
| 34 | 5 and 20 and 21 | 28 |
| 35 | 5 and 20 and 21 and 27 | 2 |
| 36 | 5 and 21 and 27 | 5 |
| 37 | 5 and 21 | 62 |
| 38 | 5 and 14 and 17 | 303 |
| 39 | or/28-38 | 3381 |
| 40 | Animals/ not (Animals/ and Humans/) | 1532753 |
| 41 | 39 not 40 | 3374 |
| 42 | remove duplicates from 41 | 3328 |
