# Supplementary material for: The approaches, theories, models, frameworks, and methods in designing toolkits to support healthcare providers in health behaviour change: A scoping review protocol
Source: PLoS One. 2026 Jun 1;21(6):e0349867. doi: 10.1371/journal.pone.0349867 (PMC13225628; doi:10.1371/journal.pone.0349867)
Supplement: S3 File — (DOCX) [file pone.0349867.s003.docx]

### **Supplementary file 3: Data extraction instrument**

The data extraction instrument has been adapted from the JBI template source of evidence details, characteristics, and results extraction instrument.

| **Scoping Review Details** | |
| --- | --- |
| Title: |  |
| Study objective/s: |  |
| Study question/s: |  |
| **Inclusion/Exclusion Criteria** | |
| Population |  |
| Concept |  |
| Context |  |
| Types of evidence source |  |
| **Evidence source Details and Characteristics** | |
| Citation details (e.g., authors/date, title, journal, volume, issue, pages) |  |
| Country |  |
| Context |  |
| **Details/Results extracted from source of evidence** (in relation to the concept of the scoping review) | |
| Toolkit name |  |
| Toolkit aims/purpose |  |
| Healthcare provider audience (e.g., primary care physicians, nurses, etc.) |  |
| Patient population targeted |  |
| Health behaviour (e.g., smoking, physical activity) |  |
| Toolkit mode of delivery (e.g., web-based, pamphlet, etc.) |  |
| Stages of toolkit development and implementation |  |
| Co-production/knowledge user engagement |  |
| Stakeholders/knowledge users engaged (e.g., HCPs, patient partners, subject experts) |  |
| Role of stakeholders (e.g., intervention design, material development, content creation) |  |
| Method of co-production/stakeholder involvement (e.g., focus groups, stakeholder panel) |  |
| Method of stakeholder/knowledge user identification |  |
| Recommendations from stakeholders and how they were addressed |  |
| Learning theory and/or behaviour change theory |  |
| Theories, Models, and/or Frameworks applied |  |
| How theories, models, and/or frameworks were applied |  |
| Toolkit componentry |  |
| Tailoring for population (e.g., age considerations, cultural adaptations, clinical needs (e.g., for patients with more severe disability)) |  |
| Facilitators |  |
| Barriers |  |
| Methods for cascading toolkit |  |
| Methods of measurement/evaluation (e.g., knowledge increase, use, behaviour change) |  |
| Outcomes (e.g., effectiveness) |  |
| Recommendations provided/lessons learned |  |
